# Augmented Humanity: A Systematic Mapping Review

**DOI:** 10.3390/s22020514

**Published:** 2022-01-10

**Authors:** Graciela Guerrero, Fernando José Mateus da Silva, Antonio Fernández-Caballero, António Pereira

**Affiliations:** 1 Departamento de Ciencias de la Computación, Universidad de las Fuerzas Armadas ESPE, Sangolqui 171103, Ecuador; rgguerrero@espe.edu.ec; 2 Instituto de Investigación en Informática de Albacete, 02071 Albacete, Spain; Antonio.Fdez@uclm.es; 3 Computer Science and Communication Research Center, School of Technology and Management, Polytechnic of Leiria, 2411-901 Leiria, Portugal; fernando.silva@ipleiria.pt; 4 Departamento de Sistemas Informáticos, Universidad de Castilla-La Mancha, 02071 Albacete, Spain; 5 CIBERSAM (Biomedical Research Networking Centre in Mental Health), 28029 Madrid, Spain; 6 Information and Communications Technologies Unit, INOV INESC Innovation, Delegation Office at Leiria, 2411-901 Leiria, Portugal

**Keywords:** systematic mapping review, augmented humanity, wearable computing, mixed reality, human–robot interaction, smart devices

## Abstract

Augmented humanity (AH) is a term that has been mentioned in several research papers. However, these papers differ in their definitions of AH. The number of publications dealing with the topic of AH is represented by a growing number of publications that increase over time, being high impact factor scientific contributions. However, this terminology is used without being formally defined. The aim of this paper is to carry out a systematic mapping review of the different existing definitions of AH and its possible application areas. Publications from 2009 to 2020 were searched in Scopus, IEEE and ACM databases, using search terms “augmented human”, ”human augmentation” and “human 2.0”. Of the 16,914 initially obtained publications, a final number of 133 was finally selected. The mapping results show a growing focus on works based on AH, with computer vision being the index term with the highest number of published articles. Other index terms are wearable computing, augmented reality, human–robot interaction, smart devices and mixed reality. In the different domains where AH is present, there are works in computer science, engineering, robotics, automation and control systems and telecommunications. This review demonstrates that it is necessary to formalize the definition of AH and also the areas of work with greater openness to the use of such concept. This is why the following definition is proposed: “Augmented humanity is a human–computer integration technology that proposes to improve capacity and productivity by changing or increasing the normal ranges of human function through the restoration or extension of human physical, intellectual and social capabilities”.

## 1. Introduction

Humans are increasingly dependent on technology. Technology has changed not only humans’ behavior and values but also the way they think, communicate and act [1]. However, recent scientific discoveries and inventions have demonstrated that technology is also beginning to modify human capabilities, pushing them beyond their natural limits [2,3,4]. With the advance of technology, the interaction between humans and machines has been “improved”, “augmented” or even “redesigned” [5,6,7,8]. This has made it not only interesting and intriguing but also viable and arising as a serious concept of scientific research and development [9,10]. A term related to this technology advancement is Augmented Humanity (AH).

The term AH was coined in 2010 at the Internationale Funk Ausstellung conference [11,12], indicating that different devices which at first glance seem unconnected to each other will in the future offer a convergence between technologies and devices that aim to interact naturally with the user. The Isobar Trend Report [13] introduces AH as technologies that can work in harmony with humans in ways that enrich life, enhance the human experience and drive sustainable progress for the benefit of people, which will involve investing and engaging underserved audiences in the process. Working together, human and artificial agents must learn and bring these terms into close collaboration between human and artificial agents. These dilemmas are largely due to the differences between human and artificial capabilities and potentialities, and the resulting tensions in their collaboration [14,15,16]. Some researchers directly interchange the terms AH and augmented reality (AR). For instance, when AR is deeper and augments the human being, it is called an augmented human [17]. Another article exposes AR as a means to create an augmented human [18]. AH is a discipline that is linked to AR, but the difference between both is not clear [19]. However, these concepts can be clarified with the example of glasses, which can be AR or AH. When the glasses complement the view, then this will be AH; if the glasses have an external functionality, for example to perform a calculation, then it is AR.

Hence, the definition of AH is currently not concrete enough, because there are several “definitions” that are not interconnected one to another. Therefore, this research presents some of the definitions obtained from the analysis of works related to the term AH. Moreover, as many works indicate that they are based on AH and have proposed devices, systems, prototypes, etc., this paper fills the need to establish a systematic mapping review for a better understanding of AH. A systematic mapping review is a type of review that allows the contextualization of systematic literature reviews within a broad literature and the identification of gaps in scientific literature. It is a review that seeks to identify not only the results of the works but also the existing links between the works carried out [20,21,22]. Therefore, the aim of this paper is to offer a systematic mapping review in order to (1) obtain a precise definition of AH to clarify the concept and use of AH and that it is not misused and (2) list the potential areas of application of AH.

This paper is structured as follows. In Section 2, we present the systematic mapping review methodology, research questions, study search strategy, data extraction and analysis and data collection efforts, providing the answers to the research questions posed. In Section 3, we discuss the results obtained from the research questions. Finally, in Section 4, we present the main conclusions of the paper and the risks that may have affected the quality of the study and how they have been mitigated or avoided.

## 2. Materials and Methods

This section introduces information on the applied research methodology, including the research protocol related to the preliminary research questions, search strategy and selection criteria. It also includes inclusion and exclusion criteria used in the research and the applied search process.

### 2.1. Research Methodology

Regarding the research methodology, in the first instance, it is necessary to mention the existing interests and objectives intended to carry out the systematic mapping review. The systematic mapping review study has been reinforced with the Preferred Reporting Items for Systematic Reviews and Meta-Analysis (PRISMA) statement [23] in conducting and reporting our review, ensuring a holistic and unbiased sampling of all published and peer-reviewed articles that are relevant to the proposed topic.

In order to achieve the proposed objectives, this research work has been structured in the following steps: The first step in this research is to obtain, through a broad analysis of the literature, summaries of different works (publications) that have utilized AH as a solution to their proposals.In this regard, the search terms employed were “augmented human”, ”human augmentation” and “human 2.0”. The second step is to obtain a trend through analysis of AH research over time. The third step is to obtain a definition that centralizes the characteristics of AH as expressed in the related works. The fourth step is to expose subthemes or research areas in AH for future research to work on from the present systematic mapping study.

**Figure 1:**
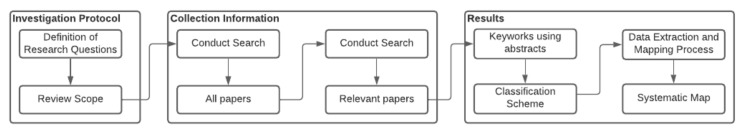
Process followed to obtain the systematic mapping review on AH [24].

The objectives mentioned are presented in Figure 1 as a process to follow in the systematic mapping review of this work. The process consists of three phases. (1) The subject of the research work to be dealt with is defined, the objectives to be met are identified and then the preliminary research questions to be answered throughout the development of this work are posed. (2) The collection of the information presented by the relevant documents found after the execution of the search chains. (3) Finally, in phase 3, the results of the keywords obtained from the relevant articles are presented. After performing the above phases, the systematic mapping is formed with the existing literature in order to answer the questions posed in the research.

### 2.2. Research Protocol

Knowing the topic of study to be addressed (in this case, AH) and the objectives established, this phase proposes preliminary research questions, search strategy and selection criteria, including an overview of the selected papers in terms of their publication over time [24,25]. Four research questions (RQ) have been defined for the present study, focused on obtaining information on the evaluation of AH applications in relation to time, proposed definitions of AH, main areas of interest in which the term AH is used, and exposure of new AH trends:

- RQ1: How many articles have been published on AH? Is there any relationship between the works that have been published on AH and the years of publication? This research question focuses on studying the number of works in relation with time on which they have been published in order to verify if there is a temporal trend of growth in publications related to AH.
- RQ2: What definitions of AH do these present and what new definition can be obtained? From this context, it is sought to determine the number of articles that have been published on AH with the aim of providing a common definition of the AH topic.
- RQ3: What areas of knowledge have been covered through works on the theme of AH? What are the most relevant areas of knowledge related to the subject matter? At this point, it is interesting to study in what areas of research the proposal has been developed and what are the most relevant areas of knowledge in which solutions are proposed with AH. In addition, it is important to obtain information on what other technologies have been able to converge with the proposed theme.
- RQ4: What future lines of work are presented as subthemes of AH? Finally, it is necessary to know what the authors propose as future research trends, as well as to determine the relevant characteristics of these works. This will allow determining the similarities between the features of those trends and to include them as contributions of the current systematic mapping review of literature.

### 2.3. Collection Information

For this study, the analysis of exclusion and inclusion of articles is performed by selecting customized search strings in indexed databases such as IEEE, ACM and Scopus. The filtering of information is done manually using inclusion and exclusion criteria based on a complete set of evaluation guidelines as established in the scientific rigor and industrial relevance [26].

#### 2.3.1. Conducting the Research

To help building the search terms, a set of key documents that are directly related to the proposed research questions was identified as a first step. Relevant synonyms were identified, and variations of the keywords were considered and added to the search. Data from the last 10 years from three databases were used to formulate the search chain: ACM, IEEE and Scopus. These databases were selected based on previous experience and advice [27]. Furthermore, Scopus has been added, since it is considered the largest database of abstracts and citations [28]. The last 10 years were taken in relation to the date the term AH was coined [11,12]. The search strings for the databases are shown in Table 1.

The information resulting from the search string shown in Table 1 has been extracted from articles in scientific journals, conferences and journals. The search of the string was carried out on 15 June 2021. Table 2 presents the numerical results obtained for each database.

#### 2.3.2. Relevant Papers

Once the articles had been retrieved from the databases, according to our search protocol, the next step was to examine their relevance. The first phase of this process consisted of examining the relevance of the articles based on their titles. Retrieved articles whose titles were relevant to our study were discarded.

#### 2.3.3. Inclusion and Exclusion Criteria

If a large number of studies are obtained, many of these may be noise or there may be duplication of works among databases [29]. In order to reduce the number of results that were obtained (16,914) by discarding duplicates and noisy data the process is carried out individually.

The following inclusion criteria were applied to titles and abstracts:

I1. Papers that focus on the subject of AH.
I2. Papers published between 1 January 2009 and 31 December 2020.

The following criteria were considered for the exclusion of documents:

E1. Studies that present non-peer reviewed material.
E2. Studies not published in English.
E3. Non-formal documents that do not have a proven scientific basis.

#### 2.3.4. Keywords

The relevant research articles from the literature were selected by following a usual process [30]. The process consisted of extracting some keywords and concepts that reflect the contributions from the abstracts of the articles. We then proceeded to read each article in detail. When it was observed that the article did not fit, it was discarded.

#### 2.3.5. Data Extraction and Mapping Process

In this stage of the systematic mapping process, information was extracted from the research articles for meta-analysis and to address the research questions. A total of 10 data items were extracted from each article, as shown in Table 3. In the first six items, basic information about the article was extracted, including the article’s year of publication, title, author(s), type of publication, etc. Items 7 and 8 extracted data about the areas of knowledge and categories in which the articles are classified; this is because WoS performs significantly better than Scopus in terms of the accuracy of its journal ranking system [31,32]. In item 9, the relevant characteristics were extracted after detailed reading of the articles, and finally, item 10 is about the abstract of each article.

## 3. Results

**Figure 2:**
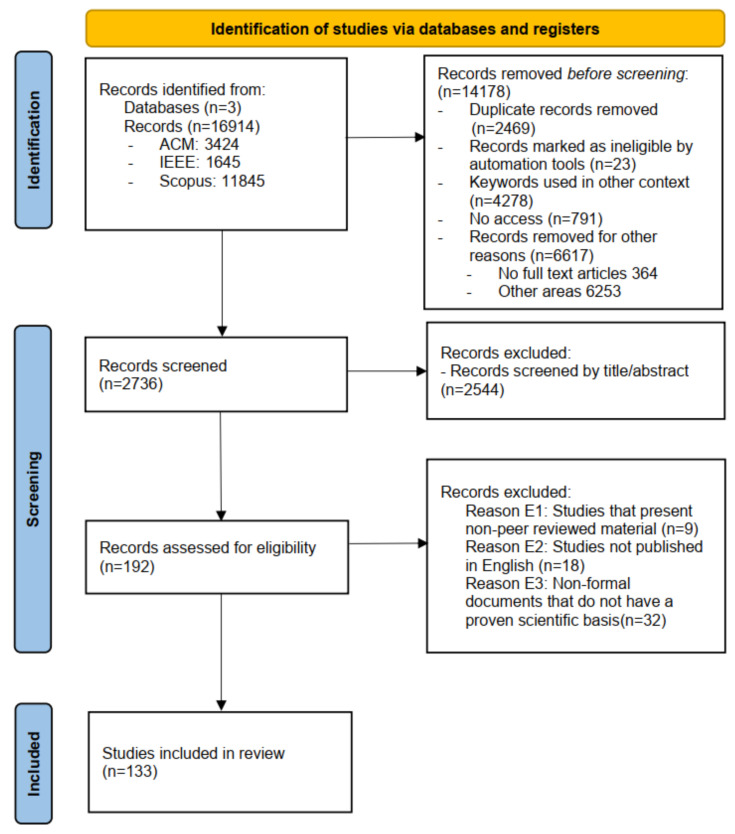
Process followed to obtain the AH systematic mapping.

After applying the search strings, 16,914 articles were retrieved from the three selected databases (see Table 2). The search process and the inclusion and exclusion criteria were applied to these data, resulting in a total of 133 articles (see Figure 2) that were eligible for analysis. This process was performed independently by two researchers who are coauthors of this review, allowing to cross-validate all the cases where there were doubts about the information. Finally, the complete content of the 133 articles was read independently by the authors to make the final decision on including or excluding them for the purpose of this research.

The PRISMA flowchart [23] (see Figure 2) consists of three phases. (1) Identification—search for related papers using the proposed search string in the different databases mentioned above. Duplicate articles, articles whose text is illegible (e.g., scanned), keywords that are not used at all or very little, additional, the context is not focused on the applicability of this research, articles that could not be accessed, articles that are not complete and articles focused on other areas are eliminated (e.g., geosciences, astronomy and astrophysics, chemistry, evolutionary biology, history, etc.). (2) Screening—phase in which the inclusion and exclusion criteria are applied to the different related works obtained by the identification phase. (3) Inclusion—phase in which the inclusion and exclusion criteria are applied to the different related works obtained by the identification phase. Here, we have eliminated those articles that have been eliminated after evaluating the title, abstract and exclusion criteria.

### 3.1. RQ1: How Many Articles Have Been Published on Augmented Humanity? Is There Any Relationship between the Works That Have Been Published on AH and the Years of Publication?

Table 4 offers the 133 scientific articles selected in this study, showing the databases where it was found and the publication year.

**Figure 3:**
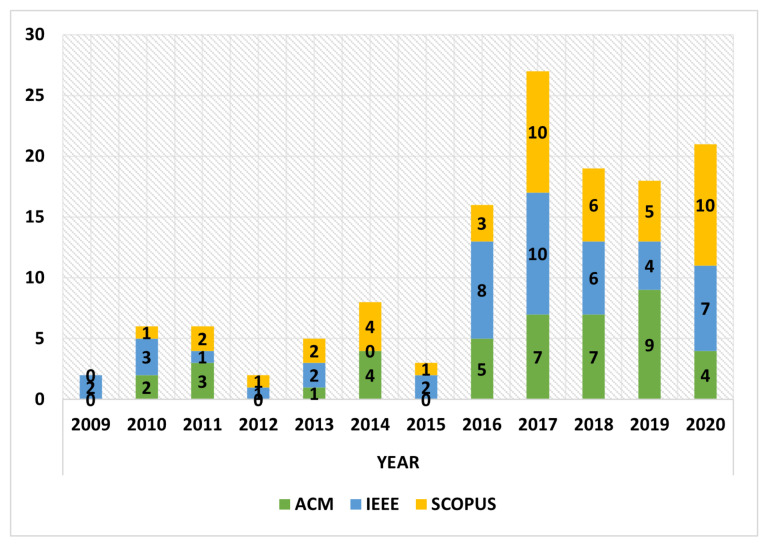
Total number of articles published per year that mention AH.

In order to know if there is any relationship between the articles obtained and the time line in which they have been published, a graph has been made (see Figure 3) with the 133 articles published, where it can be seen that there is a significant growth in the year 2017 and a stabilization of the number of articles in 2018 and 2019. For the year 2020, there is an increase of articles compared to the two previous years, 21 articles have been selected. Nonetheless, it is still a lower number than the articles of the year 2017 (27).

Figure 3 shows the number of studies identified within the years 2009–2020. Between the years 2009 and 2015, it can be observed that the AH theme was in development, with highs and lows of articles. A significant rise in the number of publications in those databases can be observed in the years 2016 and 2017. From 2009 to 2020, there is an increase in the number of studies on AH.

As part of the answer to RQ1, we have also considered the number of papers obtained in relation to the databases searched, which, according to Figure 3, contain similar publication numbers by year (after removing duplicates), leaving the IEEE as the database where a larger number of articles dealing with the subject of AH are indexed with 46 articles, followed by Scopus with 45 articles and ACM with 42 articles.

### 3.2. RQ2: What Definitions of AH Do These Present and What New Definition Can Be Obtained?

The AH definitions that were obtained are shown in Table 5, where only 17 articles out of 133 have been extracted, as they are the only ones that have provided definitions of AH.

In order to obtain a definition of AH that contains the context of the 17 articles that already define the subject, we considered the number of citations regardless of the fact that most of the articles were published in the last 11 years, justifying the need to consider the number of views of each one of them as well, as shown in Table 6.

Finally, the four articles whose definitions attempt to encompass the entire context of AH were selected. From these articles, those with the greatest number of views and citations were selected [35,36,87,110,116], allowing us to propose the following definition:

*Augmented humanity is a human–computer integration technology that proposes to improve capacity and productivity by changing or increasing the normal ranges of human function, through the restoration or extension of human physical, intellectual and social capabilities.*

The justification of this new formal definition of AH is detailed next as regards the constituent parts of which it is made up:

- *Technology*: In accordance with [163], AH is described as a technology in relation to its “inputs” and “outputs”, where the inputs of technology are knowledge, resources and labor, while the outputs are material culture and modification of the environment. Since technology is the immediate point of contact between people and environment, the consideration of the term technology acknowledges the limiting and shaping functions of the physical and social environment [164]. Furthermore, the content of technology can be conceived in terms of knowledge, applications or norms [164,165].
- *Human–computer integration*: The definition also expounds on the concept of human–computer integration, which is the symbiotic partnership or relationship in which humans and software give rise to patterns of behavior that must be considered holistically [35,166].
- *Improvement of capacity and productivity*: Augmentation is the most common term in the interdisciplinary research community that focuses on interactive digital extensions of human capabilities [95]. The related concepts of augmented human and human 2.0 [167] refer to technologies that augment human productivity or capability or add to the human body or mind [168]. Human augmentation will serve the user by providing essential and timely information for common tasks [95] such as working, driving and so on.
- *Change or increase of normal ranges of human function*: Human augmentation products and/or applications can be made for anyone, from healthy users who wish to improve their human capabilities to users who face temporary or permanent disabilities, physical disabilities or hazardous situations requiring their use [169].
- When referring to AH as *improving the physical, intellectual and social capabilities* of human beings, we allude to all possibilities that are framed in each main component of AH. More concretely:
  - *Augmented physical capabilities* are achieved through the interpretation of the augmented senses and the actions they produce. Vision, taste, touch, smell and hearing can be physically augmented.
  - *Augmented intellectual capacities* are achieved through the acquisition of knowledge, cognitive processing and reasoning. Numerical aptitude, verbal comprehension, perceptual speed, inductive reasoning, deductive reasoning, spatial reasoning, abstract reasoning, memory, will and so on can be augmented intellectually.
  - *Augmented social skills* are considered through the interpretation of basic and complex social skills. Empathy, emotional intelligence, assertiveness, listening skills, ability to communicate emotions, ability to define a problem and evaluate solutions, negotiation, presenting oneself and many more can be socially augmented.

### 3.3. RQ3: What Areas of Knowledge Have Been Covered through the Work on the Theme of Augmented Humanity? What Are the Most Relevant Areas of Knowledge Related to the Subject Matter?

**Figure 4:**
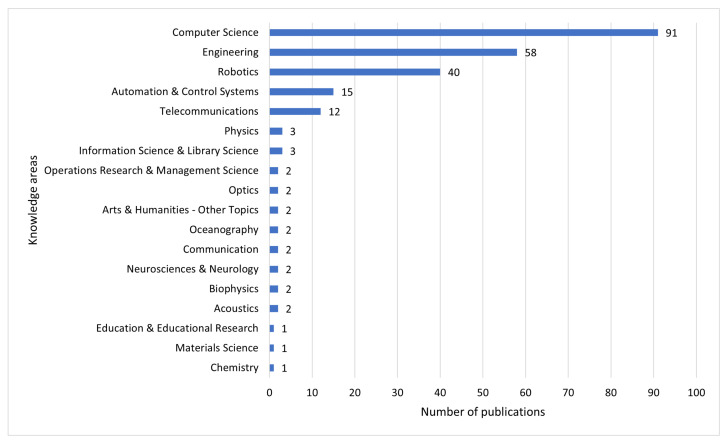
Web of Science research areas assigned to the 133 publications.

The total number of knowledge areas for the 133 articles is 18. In Figure 4, it can be seen that there are five relevant areas in which the selected research works are focused: computer science (91), engineering (58), robotics (40), automation and control system (15) and telecommunications (12).

**Figure 5:**
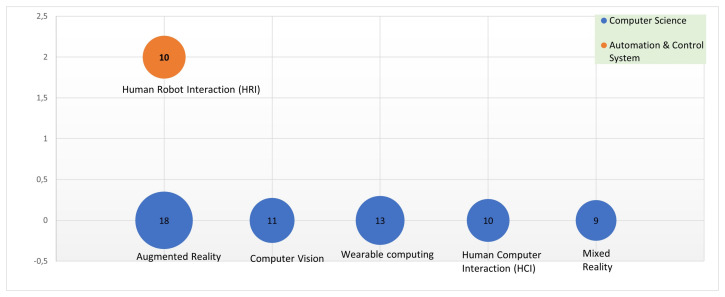
Number of keyword repetitions per area.

Of the 133 articles selected, the repetition rate of the keywords was extracted, the most repeated are: augmented reality; wearable computing; computer vision; human–robot interaction and mixed reality. In relation to the various areas of knowledge, it is clear that most of the articles selected are related to technology, of which the most relevant categories are: (i) computer science; (ii) engineering; (iii) robotics; (iv) automation and control systems; and (v) telecommunications. Figure 5 presents the relevant domains of AH in relation to the number of keyword repetitions per area. There are two relevant fields of study (*y*-axis) that are clearly outstanding: computer science and automation and control systems.

**Figure 6:**
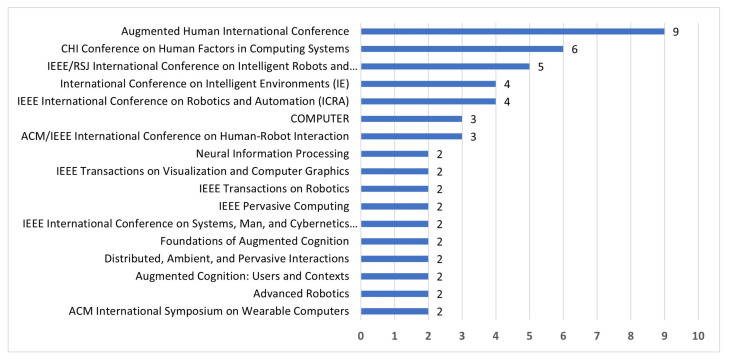
Publication venues of research articles.

Besides the obtained research areas, we have also studied the conferences (peer-review), magazines and journals in which the research works have been published. There are 18 venues whose publication repetition index of the 133 articles is higher than 1. The other publications in conferences, magazines or journals have been omitted. The most relevant publications are depicted in Figure 6, where three publication venues of relevant research papers stand out: Augmented Human International Conference (9 publications), CHI Conference on Human Factors in Computing Systems (6 publications) IEEE/RSJ International Conference on Intelligent Robots and Systems (IROS) (5 publications) and IEEE International Conference on Robotics and Automation (ICRA) (4 publications).

**Figure 7:**
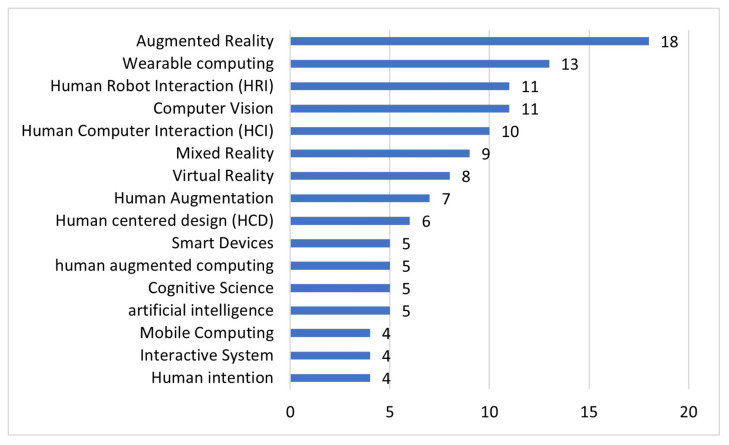
Top Keywords with repetition.

Finally, the index terms of each one of the 133 selected articles are considered for this study. The total number of keywords is 200. The keywords selected for the grouping are those that have been repeated more than twice. Figure 7 shows the major topics covered (top ten) with the respective number of repetitions. Augmented reality is the most popular one, with 18 citations in the keywords of the scientific articles. The five following topics with the highest repetition rate are: wearable computing (13), human–robot interaction (11), computer vision (11), human–computer interaction (10) and mixed reality (9).

It is curious that the keyword human augmentation has a repetition rate of 7 times; this is because although in the titles and text of the publications the word exists, it does not exist as a keyword proposed by the authors. Finally, of the number of publications obtained, it is necessary to mention that they have been published in congresses and conferences with a high impact factor.

### 3.4. RQ4: What Future Lines of Work Are Presented?

We decided to present a compilation of the proposals for future works from the first five positions of the research (computer science, engineering, robotics, automation and control systems and telecommunications) according to Figure 4 (see Supplementary Materials Table S1). It was also considered to express in Supplementary Materials Table S1 the theme of the research work a brief description of the objective of the work and the proposals presented for future work. The reason why 37 articles are shown in Supplementary Materials Table S1 and not 133 is because we have taken those articles that have been cited in the key words by the authors: wearable computing, computer vision, human–robot interaction and mixed reality.

**Figure 8:**
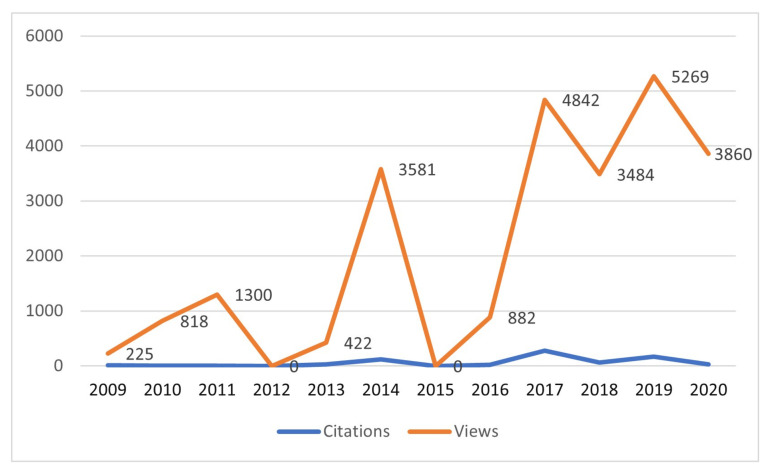
Number of article citations and article views per year of publication.

Table 7 and Figure 8 expose the works related to the theme of AH until 2020. These works are focused on different areas. However there are works that somewhere in its structure mention “augmented humanity” as a feature of the development of the authors.

## 4. Discussion

This work has introduced a systematic mapping review of the definitions of AH as well as the published research that has been developed under this theme and that have been published in recent years (2009–2020). The results obtained are important and show a global vision of the current state of knowledge in this area. In relation to the definitions obtained about AH, it was not possible to obtain a single formal one from the analysis of the different proposals studied in the analysis of the related works. The results that we retrieved provide a global perception of the state of knowledge and study of the area on the subject of AH. A first certainty of this systematic review was that more research is still needed to characterize, organize and classify the AH terminology within its respective fields of study and translate it into other scientific contributions based on the new definition provided in this research paper. Indeed, our definition can be used not only in technology but in any field looking for human support through human–computer integration.

It is important to emphasize that in relation to the publication venues, the great majority of works were disclosed in conferences (peer-review) and in journals of high impact factor. As for the temporal evolution, there has been the tendency of the slight growth (4.5 publications in average per year) of publications until the year 2016, when it increased (16 publications), and in the year 2017, the peak of the curve was observed; then, for the years 2018 and 2019, the publications between 19 and 18 stabilized. For the year 2020, 21 publications were published up to December 31, compared to the two previous years the related articles went up. The increase is not equal or higher to year 2017. One of the possible reasons may be to the fact that in year 2020 the COVID-19 virus SARS-CoV-2 spread worldwide, causing a decrease of articles published. However, in recent years, there has been an increase in publications in general terms, and this may be an indicator that the AH terminology is gradually becoming a focus of interest to the research community. In the articles reviewed in this paper, it is appreciated that there are several terms in which AH exists. However, it is not directly linked to AH terminology; possible reasons could be lack of knowledge of a concrete definition, publications focus on the specific field and lack of knowledge of a classification of AH.

The results obtained in the present review of systematic mapping on definitions of AH show that in some publications a macro concept of definition is emitted, without this one having been defined by means of a work similar to the present one. In other publications, even, it is only mentioned that some thematic is being done with AH, giving no notion about what it is. This systematic mapping review has introduced a series of scientific publications that define AH from different approaches or perspectives, some of these depending on the area in which the research is focused. All the existing definitions may cause confusion to the reader, hindering the understanding of an exact definition of AH. That is why four research questions were posed to resolve doubts about the number of related works that have used the term AH until December 2020, these being 16,914. However, they did not present a concrete definition on the subject. After applying inclusion and exclusion criteria, only 17 high impact related works presented definitions in which there were significant differences in approach. An analysis of the context of each proposed definition was carried out and we provided our proper definition.

In the third question posed, the knowledge areas relevant to the subject of AH according to WOS, in which AH causes greater impact, being the five areas of study: computer science, engineering, robotics, automation and control systems and telecommunications, showing that AH is an interdisciplinary terminology. In this regard, only a few papers from the significant number of articles mentioning the term AH in the diverse knowledge areas have provided some definition of AH. This has probably lead to confusion about the orientation and purpose of the term. In fact, the term can be used in many areas of knowledge because AH encompasses any technological aspect supporting human beings, whether physically, intellectually or socially. However many researchers did not find it necessary to properly label their conception of AH.

In the fourth research question, the related works closer to the topics about the repetition rate of the most used terms are analyzed and characteristics are extracted and proposals for future works are exposed, leaving open research proposals. These research proposals are focused on the areas of computer science (38.23%), engineering (24.36%), robotics (16.80%), automation and control systems (6.30%) and telecommunications (5.04%). There are other areas where the term is also used, for example: information science and library science, physics, neuroscience and neurology, optics, etc. Regardless of the knowledge areas and keyword repetition rate that indicate an increased use of AH terminology, probably not all users will fully accept human augmentation, which may have caused noise or bias suggestions with respect to augmented human capabilities. In other words, some constraints in defining AH may have arisen from user perception, considering the three main components suggested by our definition. (i) Physical capabilities: users may be slow to accept a physical augmentation, which may be due to habit, resistance to change, etc. (ii) Intellectual capabilities: users may take time to learn to manipulate or augmented humanity applications. This may occur for different reasons such as cognitive-affective management, either training time, prior knowledge of manipulating technological interfaces, etc. (iii) Social capabilities: users do not tend to have a regular pattern; by nature, they are changeable in their tastes, behaviors, feelings, etc.

The keywords with the highest repetition rate were also exposed, being the six main ones: augmented reality (14.4%), wearable computing (10.4%), human–robot interaction (8.8%), computer vision (8.8%), human–computer interaction (8%), mixed reality (7.2%) and others (42.4%). These data indicate the terminology with greater application used in AH research, clearly indicating an orientation with greater experience on the knowledge of what AH means. However, the 42.4% of most used keywords could reflect a lower AH approach, so we think that with the definition proposed in this document these percentages could be raised, opening or positioning those keywords to a greater extent. Yet, the term AH should not be confused with augmented reality, virtual reality and/or mixed reality. Although these can be the tools and/or means for implementing AH applications, AH may use any other means or technology capable of designing applications that contribute to improve or enhance the physical, intellectual or social capabilities of human beings.

Throughout the development of this work, three possible risks that could have affected the systematic mapping review have been considered:

- The first possible risk [170], is the bias of selective information because the analyses of statistically significant differences are published more frequently. To minimize this risk, three scientific databases have been used as sources for the search process: IEEE, ACM and Scopus. These provide a complete list of articles covering the different aspects of this systematic mapping review. To avoid affecting the validity of the present study, literature from theses, reports, etc., has been excluded, since these types of documents do not have as rigorous of a review process as that of journals.
- The second possible risk that could affect the study is selection bias. However, for this risk, the inclusion and exclusion criteria were clearly described.
- The third possible risk that could have affected this research is related to the possibility of classifying a study in different ways. This risk was mitigated by the support and joint work of four researchers, so that when a researcher presented some doubt or there would be discrepancies between the classifications, it was discussed with the working group, allowing to improve the quality of the analysis.

This systematic mapping review also has some limitations. Although the authors attempted to identify as many articles as possible, studies not published in English were excluded from the review, so that some other AH cues may have been missed. Another limitation of our review has to do with ethical issues, as we did not study this aspect in the articles searched. All AH applications are required to adopt ethical commitments since they deal with human beings. For example, they must take care of the privacy of personal data.

In relation to the proposed new definition of AH, there may be a latent limitation due to the fact that the term is novel and AH is only taking its first steps. This means that it is likely that the use of our definition will not land until more researchers engage with the terminology. In addition to this, it may lead to self-interested negativity in the use of the AH definition, as AH is a term that can be used cross-cuttingly in many areas of knowledge and may give way to a desire to formulate more specific definitions focused on each field. Another limitation for the use of our definition is that the studied works emerged in the years where AH was only informally declared. However, in the course of time, studies using AH will continue to be proposed and developed and there may be a breaking point in which it will be necessary to revisit the definition and add additional features.

Finally, it is proposed as future work to carry out a classification or taxonomy of the areas in which the different research approaches in which AH is applied are presented. Additionally, future works of different researchers are presented, whose approaches are based on developing applications, prototypes or research in augmented reality, wearable computing, computer vision, human–robot interaction (HRI), mixed reality and human–computer interaction (HCI). For example, four possible augmented humanity works are cited: (i) Perform augmented writing using a transmissive head-mounted display, such as Microsoft’s HoloLens, where only one user can experience the effects without showing anything in real space. (ii) Develop representative wearable 2.0 applications for health care including chronic disease monitoring, elderly care, medical and health care institution, smart training for athletes, emotion care, etc. These applications can be virtual reality/augmented reality applications based on smart wearables. (iii) Develop a theoretical framework that provides a structural basis for exploring intelligence augmentation. iv) Improve interaction metaphors (image, resolution, flight times, display field of view, etc.) of a drone for remote location visualization.

## Figures and Tables

**Table 1:** Search strings.

Database	Search String
ACM	[[[Publication Title: augmented] AND [Publication Title: human]] OR [[Publication Title: human] AND [Publication Title: augmentation]] OR [Publication Title: human +] OR [Publication Title: “2.0”]] AND [[[Abstract: augmented] AND [Abstract: human]] OR [[Abstract: human] AND [Abstract: augmentation]] OR [Abstract: human +] OR [Abstract: “2.0”]] AND [[[Keywords: augmented] AND [Keywords: human]] OR [[Keywords: human] AND [Keywords: augmentation]] OR [Keywords: human +] OR [Keywords: “2.0”]] AND [Publication Date: (01/01/2009 TO 31/12/2020)]
IEEE	(((((((((”Document Title”:augmented human) OR “Document Title”:human augmentation) OR “Document Title”:human 2.0) OR “Author Keywords”:augmented human) OR “Author Keywords”:human augmentation) OR “Author Keywords”:human 2.0) OR “Abstract”:augmented human) OR “Abstract”:human augmentation) OR “Abstract”:human 2.0)
Scopus	TITLE-ABS-KEY((augmented AND (human OR humanity)) OR (human AND (augmentation OR “2.0”))) AND (LIMIT-TO(SUBJAREA, “COMP”)) AND (LIMIT-TO (LANGUAGE, “English”))

**Table 2:** Database search results.

Database	Numerical Studies	Total Studies
ACM	3424	
IEEE	1645	16,914
Scopus	11,845	

**Table 3:** Extracted data items.

#	Data Item	Description
1	Identifier	Paper identification
2	Year	Publication year
3	Title	Title of the paper
4	Authors	Authors of the paper
5	Publication channel	Channel for publishing the paper
6	Keywords	Keywords of the paper
7	Research area	Research areas taken from Web of Science
8	Category	Research categories taken from Web of Science
9	Paper contributions	Main contributions of the paper
10	Summary	Our own summary or abstract of the paper

**Table 4:** Articles that contain the search keywords in their title.

Article	Database	Year	Article	Database	Year	Article	Database	Year
[33]	IEEE, Scopus	2020	[34]	IEEE, Scopus	2019	[35]	ACM, Scopus	2020
[36]	Scopus	2014	[37]	Scopus	2019	[38]	ACM, Scopus	2011
[39]	Scopus	2020	[40]	Scopus	2019	[41]	IEEE, Scopus	2018
[42]	IEEE, Scopus	2016	[43]	IEEE	2017	[44]	IEEE, Scopus	2016
[45]	ACM, Scopus	2018	[46]	ACM, Scopus	2018	[47]	IEEE, Scopus	2012
[48]	Scopus, ACM	2020	[49]	IEEE	2019	[50]	Scopus, IEEE	2012
[51]	IEEE, Scopus, ACM	2020	[52]	IEEE, Scopus	2010	[53]	Scopus	2013
[54]	ACM, Scopus	2018	[55]	Scopus, ACM	2011	[56]	ACM, Scopus	2010
[57]	Scopus, ACM	2015	[58]	IEEE, Scopus	2013	[59]	Scopus	2016
[60]	IEEE, Scopus	2017	[61]	Scopus, IEEE, ACM	2018	[62]	Scopus, ACM	2020
[63]	Scopus, IEEE	2018	[64]	ACM	2020	[65]	ACM, Scopus, IEEE	2019
[66]	IEEE, Scopus	2020	[67]	Scopus, ACM	2013	[62]	ACM, Scopus	2016
[68]	IEEE, Scopus	2020	[69]	Scopus, IEEE	2020	[70]	Scopus, IEEE	2017
[71]	Scopus	2017	[72]	Scopus, ACM	2019	[73]	Scopus	2020
[74]	IEEE, Scopus, ACM	2016	[10]	IEEE	2013	[75]	Scopus, IEEE	2010
[76]	ACM, Scopus	2018	[77]	ACM, Scopus	2017	[78]	IEEE, Scopus	2019
[79]	ACM, Scopus	2016	[80]	IEEE, Scopus, ACM	2016	[81]	ACM, Scopus	2014
[82]	IEEE, Scopus	2017	[83]	Scopus	2018	[84]	IEEE, Scopus	2010
[85]	Scopus	2014	[86]	Scopus, ACM	2017	[87]	Scopus	2017
[88]	ACM, Scopus	2017	[89]	Scopus	2018	[90]	IEEE, Scopus	2018
[91]	IEEE, Scopus	2018	[92]	ACM, Scopus	2016	[93]	ACM, Scopus	2014
[94]	Scopus	2014	[95]	Scopus	2019	[96]	IEEE, Scopus	2018
[97]	ACM, Scopus	2018	[98]	IEEE, Scopus, ACM	2009	[99]	IEEE, Scopus, ACM	2017
[100]	Scopus	2017	[101]	IEEE	2017	[102]	Scopus	2017
[12]	Scopus	2020	[103]	IEEE, Scopus, ACM	2016	[104]	Scopus	2020
[105]	ACM	2018	[106]	Scopus	2018	[107]	ACM, Scopus	2019
[108]	ACM, Scopus	2010	[109]	IEEE, Scopus, ACM	2015	[110]	IEEE	2017
[111]	ACM, Scopus	2019	[112]	ACM, Scopus, IEEE	2011	[92]	Scopus, ACM	2016
[113]	Scopus	2014	[114]	IEEE, Scopus	2018	[115]	ACM, Scopus	2013
[116]	IEEE, Scopus, ACM	2017	[117]	IEEE, Scopus	2020	[118]	ACM, Scopus	2011
[119]	ACM, Scopus	2017	[120]	ACM	2014	[121]	IEEE, Scopus	2018
[122]	Scopus, ACM	2016	[123]	Scopus	2017	[124]	Scopus	2011
[125]	ACM	2019	[126]	Scopus	2017	[127]	ACM, Scopus	2019
[128]	ACM, Scopus	2018	[129]	IEEE, Scopus	2015	[130]	ACM, Scopus	2017
[131]	Scopus, ACM	2020	[132]	IEEE	2017	[133]	Scopus, ACM	2017
[134]	IEEE, Scopus	2016	[135]	IEEE, Scopus	2016	[136]	IEEE, Scopus, IEEE	2019
[137]	Scopus	2020	[138]	ACM, Scopus	2014	[139]	Scopus, IEEE	2020
[140]	IEEE, Scopus	2011	[141]	ACM, Scopus	2019	[142]	ACM, Scopus	2019
[143]	ACM, Scopus	2017	[144]	ACM, Scopus	2019	[145]	ACM, Scopus	2019
[146]	IEEE, Scopus	2016	[147]	IEEE, Scopus	2010	[148]	IEEE, Scopus	2020
[149]	ACM, Scopus	2020	[150]	IEEE, Scopus, ACM	2009	[4]	ACM, Scopus	2016
[151]	Scopus, IEEE	2019	[152]	IEEE, Scopus	2017	[153]	Scopus, ACM, IEEE	2017
[154]	ACM, Scopus	2017	[155]	IEEE, Scopus	2017	[156]	ACM, Scopus	2020
[157]	Scopus, IEEE	2018	[18]	ACM, Scopus	2016	[158]	ACM, Scopus	2017
[159]	IEEE, Scopus	2020	[160]	Scopus	2014	[161]	Scopus, ACM	2020

**Table 5:** Definitions of Augmented Humanity.

Article	Definition
[119]	Augmented Humanity is the improvement of traditional human–human and human–machine interaction by augmenting humans with portable technology and developing new user interfaces.
[105]	Computers augmenting humans enable instant information access. Yet, interactions between these two sides, the augmented human within an augmented world, are still different from human–human and from device–device interactions.
[83]	Augmented human is a human whose physical, intellectual and social ability are enhanced by the augmented/virtual reality and the smart ICT technology.
[100]	Google CEO, Eric Schmidt, has called this “augmented humanity”, where networked devices “just work and understand autonomously” [11].
[87]	Human augmentation amplifies and enhances human ability to do work. Encompasses many technologies: prosthetics, orthotics and physically assistive devices that replace missing or lost functions; exoskeletons that extend physical abilities; collaborative systems that work alongside people to fill in and complement human abilities; and socially assistive robots that monitor and motivate human work and effort.
[135]	Advanced human augmentation suggests technologies that augment human actions, senses and cognition in new, as yet unexplored ways, in order to enhance human senses, to provide assistive augmentation and to create a seamless technology environment for human interaction.
[97]	An augmented human is a person who is able to use AR effectively to expand the physical, intellectual and social abilities of the user.
[162]	Augmented humanity refers to the digital administration of the world, where the human converges with computer electronic devices and instruments, generating a natural environment for the user, where even the user is not aware of the new technologies that he is using for himself.
[36]	Augmented human refers to a research direction of enhancing or augmenting human abilities by human–computer integration.
[132]	Augmented human introduces a fundamental paradigm shift in HCI: from human–computer interaction to human–computer integration, and abilities will be mutually connected through the networks (what we call IoA, or Internet of Abilities, as the next step of IoT, Internet of Things).
[101]	Human augmentation is a deliberate act. It is a permanent or temporary bodily intervention that changes or augments otherwise normal ranges of human function.
[110]	Technologies that enhance human productivity and improve or restore capabilities of the human body or mind are an area of computing we refer to as human augmentation.
[116]	Augmenting human intellect and amplifying perception and cognition as various technologies designed to augment the human intellect and amplify human perception and cognition.
[35]	Human–computer integration (HInt) is considered a new paradigm with the key property that computers integrate closely with the user. Such integration occurs primarily at the individual level through sensory fusion, with computers providing information directly to the human senses rather than through symbolic representations and understanding the implicit and precognitive needs of the user through biosensitization. However, there is also the observation that this integration occurs at a social level, where the human being and the interface agents make a coordinated effort to achieve a common goal.
[95]	Human augmentation is an interdisciplinary field that addresses methods, technologies and their applications for enhancing sensing, action and/or cognitive abilities of a human. This is achieved through sensing and actuation technologies, fusion and fission of information and artificial intelligence methods.
[12]	AH involves augmenting humans with devices that can collect data from the individual and the individuals’ environment and transmit this data to an external device or service.
[64]	Human augmentation is an approach to enhancing and empowering human functions with information technologies utilizing robotics and sensing devices.

**Table 6:** Number of citations and readings to publications defining AH.

Article	Citations	Views	Year	Article	Citations	Views	Year
[119]	2	206	2017	[132]	1	205	2017
[105]	0	87	2018	[101]	4	1148	2017
[83]	2	1100	2018	[110]	5	2595	2017
[100]	0	10	2017	[116]	28	1173	2017
[87]	103	(*)	2017	[35]	47	1819	2020
[135]	0	233	2016	[95]	41	202	2019
[97]	2	218	2018	[12]	0	1100	2020
[162]	0	1101	2017	[64]	4	639	2020
[36]	5	24,000	2014				

(*) No information provided.

**Table 7:** Number of citations and readings of publications related to AH.

Article	Citations	Views	Article	Citations	Views	Article	Citations	Views
[108]	0	191	[145]	22	744	[159]	2	180
[98]	11	225	[142]	9	398	[78]	0	40
[84]	2	627	[111]	2	58	[66]	3	352
[61]	60	3484	[117]	6	256	[95]	41	202
[88]	1	158	[127]	11	148	[37]	15	73
[138]	104	1181	[141]	0	230	[151]	5	115
[18]	18	742	[107]	31	1247	[33]	1	149
[134]	2	140	[65]	1	202	[39]	3	1300
[160]	13	2400	[125]	13	299	[149]	5	403
[70])	17	733	[115]	31	422	[139]	0	88
[124]	2	1300	[136]	13	1333	[148]	7	724
[133]	5	231	[68]	2	95	[156]	2	493
[153]	255	3720						

## Data Availability

All data generated or analyzed during this study are included in this article. The review protocol is available on reasonable demand from the corresponding author.

## Supplementary Materials

The following are available online at: https://www.mdpi.com/article/10.3390/s22020514/s1, Table S1: Proposals for future work.
