# Supplementary material for: Augmented Humanity: A Systematic Mapping Review
Source: Sensors (Basel). 2022 Jan 10;22(2):514. doi: 10.3390/s22020514 (PMC8778398; doi:10.3390/s22020514)
Supplement: Supplementary file 1 [file sensors-22-00514-s001.zip › sensors-1494491-supplementary.pdf]

Supplementary Material S1: Proposals for future work

Article	About the paper	Future works
[53]	The paper presents a technique for mixing printed photographs with digital content with the goal of preserving the emotionally rich and affective state of photographs viewing, sharing and discussing.	The initial prototype could be enhanced by mixing the table and the display into an interactive tabletop.
[54]	The authors suggest a new method of human augmented mapping for indoor environments using only a stereo camera. The user can share information about the environment with the robot and add semantic information to the environmental map.	The authors state that the global topological map is erroneous in the User's Guidance process. Thus, they propose the two-way pose estimation method to fix it in the Robot's Map Revision process.
[55]	Smart robot glasses are proposed for human visual augmentation, which consist of multiple cameras with simple mirror optics, microphones, wireless speakers, interfacing electronics, and signal processing system. For efficient face recognition task, the bio-motivated recognition algorithm architecture is applied, and the visual saliency map is used to focus the human intention to the specific areas.	The authors propose to make the integration system of the mini camera recognition system and the mini display system as a smart robotic device for simultaneous human visual and auditory augmentation. It will be useful especially for multimodal recognition of combining video and audio information.
[56]	The authors propose a prototypical system to discover a remote or occluded scene in an intuitive way by visualizing live imagery streamed from a camera drone in a three-dimensional, exocentric context.	Improve interaction metaphors (image, resolution, flight times, field of view of the screen, etc.) of the drone for the visualization of a remote place.
[57]	The authors try to extend the discussion about the human augmentation of the mind through reasoning.	Applying augmented human mind in general is difficult because it is underlying any domain. For this, it is necessary to develop a solution to recognize some biases through natural language, but in real life many cognitive biases do not come in a very formal way, they are highly integrated in the flow of activities. Therefore, the system would have to rely much more on ubiquitous computing, using other sensors to detect cognitive biases of non-natural language.
[58]	JackIn is presented as a new human-human communication framework for connecting two or more people. With first-person view video streaming from a person (called Body) wearing a transparent head-mounted display and a head-mounted camera, the other person (called Ghost) participates in the shared first-person view. With JackIn, people's activities can be shared, and assistance or guidance can be given through other people's expertise. This can be applied to daily activities such as cooking lessons, shopping navigation, education in craftwork or electrical work, and sharing experiences of sporting and live events.	The authors propose investigate other sensing technologies such as integration with motion sensors and 3D reconstruction with depth sensors.
[59]	A prototype of an application in augmented reality that aims to simulate a food with the aim of avoiding anxiety when eating is proposed. All these simulated characteristics include vision, hearing, haptic, smell, burst, etc. and other sensations.	Implement prototypes for develop augmented Human Food Interaction techniques will help individuals control their food consumption more effortlessly without losing the pleasure of eating and have significant effects in promoting nutritional health.
[60]	A new authentication model based on physical location of a person wearing or passing by BLE beacons is conceived. A part of the overall concept - BLE Beacons based path tracking is validated - with a real implementation and critically evaluated its weak points and proposed potential improvements.	The authors propose a larger scale deployment and test scenarios in the shops of a real smart city in the mountain, i.e., a smart ski resort.

Supplementary Material S1: Proposals for future work (cont.)

Article	About the paper	Future works
[61]	A framework and research methodology are described to support instantiation of physiologically driven, adaptive AR to assess and contextually adapt to an individual's environmental and cognitive state in real time.	Researchers will continue to explore how to integrate a dynamically changing sensor/software framework into a context-aware AR platform [62-64] to augment human perceptual capabilities and improve human performance.
[65]	Proposes a new interface framework for human-computer interaction in industry that can overcome the current limitations of previous works. The framework uses a laser-writer instead of a projector which is suitable for both indoor and outdoor applications.	Conducts a usability testing to evaluate the effectiveness of our interface and further improve the proposed framework using user's feedback.
[66]	The authors present a prototype, called miBook, for edutainment by providing several types of multimedia contents and, at the same time, providing the possibility of co-creation and of interaction in real time, reshaping reality with virtual creativity.	Future work should consist in strengthening effective technological development coupled with appropriate industry cluster as well as policy development, critical to the evolutionary stages of miBook. The use of cloud computing shall allow the miBook's production in a dramatically reduced time in a processing open platform compatible with the work.
[67]	The authors introduce a prototype of a collaborative environment based on augmented reality with the aim that users can program robots with a high level of abstraction and perform collaborative tasks for large-scale production of products.	Adding tactile user interfaces to create new programs or instructions to the robots. Implement the agile change between the interfaces so that the user does not delay the tasks to be programmed.
[68]	Authors propose a Wearable 2.0 healthcare system to improve Quality of Experience and Quality of Service of the next generation healthcare system. In the proposed system, washable smart clothing, which consists of sensors, electrodes, and wires, is the critical component to collect users' physiological data and receive the analysis results of users' health and emotional status provided by cloud-based machine intelligence.	Develop representative health care applications of Wearable 2.0 that include chronic disease monitoring, elder care, medical and health care institution, intelligent training for athletes, emotion care, etc. These applications can be Applications of Virtual Reality/Augmented Reality Based on Smart Clothing included.
[69]	An application based on virtual performance augmentation (VPA) is presented, using virtual reality to give users the illusion of having better capabilities than they have when they exercise. Concluding that virtually increasing running and jumping can increase intrinsic motivation, perceived competence by increasing motivation to exercise and stay healthy.	Develop a rehabilitation-oriented virtual performance enhancement application.
[70]	Presents a mixed reality application that allows young adults with Autism Spectrum Disorder (ASD) the opportunity to practice job interviews. It uses the AR hardware sensors of the Magic Leap tool to provide users with immediate feedback on six different metrics, including gaze, blink rate, and head orientation.	Perform usability tests with users, validating detection data (e.g., blink speed, eye gaze) for people with ASD and a neurotypical population.
[71]	The researchers propose a keyboard to increase the user experience, so that they can receive sounds and also visualize graphics according to their writing.	The authors propose a method for performing augmented writing using a transmissive head-mounted display such as Microsoft's HoloLens, where only a user can experience the effects without displaying anything in real space.
[72]	The authors present a prototype lens (consisting of a pair of glasses and a high-speed synchronized projector) to focus on certain objects, while also being able to blur the vision of other objects, highlighting the use of augmented vision for this research.	Investigate more robust and flexible techniques to use the method under more relaxed conditions, such as under environment lighting.

Article	About the paper	Future works
[73]	The authors present a second-screen TV viewing agenda to explore the opportunities offered by wearable devices and appliances, such as smart glasses and head-mounted screens, to provide visual experiences for users and as a contribution to the TVX community.	Evaluate and propose constructive approaches and developments towards new devices, application opportunities and tools to visually enhance the television viewing experience.
[74]	A microscopy device to provide the user with the sensation of touch, by expanding or increasing casual and tactile sensory capabilities in micrometric environments, is presented. The device, called Magnifinder, is a fingertip-sized device that uses a spherical lens, an image sensor, and an actuator to deliver the enhanced sensations.	Performing usability and sensitivity evaluations on the perceived sensations.
[75]	A prototype for tracking behavior such as facial expressions, body movements and the mixing of different human behaviors is presented to synthesize and increase social interactions through virtual reality.	Expand the database of body expressions obtained and also focus on the different facial expressions of the human, harmonizing the movement of the entire human body.
[76]	The author presents 4 platforms to experience human-robot interaction focused on music. These platforms are: 1.- A robot capable of integrating to the sounds to play drums. 2.- A robot that plays marimba capable of communicating through music and social gestures. 3.- A robot that provides improvements in the musical experience that can respond to movements thanks to its five degrees of freedom. 4.- Wearables to improve the musical experience for people with disabilities.	The author proposes 4 platforms in which applications have been developed to improve the human-robot experience in the field of music, the author proposes to continue developing applications with the same type of approach for future work.
[77]	A physical enhancement is proposed by developing a tail to support an individual's weight, similar to a kangaroo's tail. Studies are also presented on the perceptual characteristics of the user to capture the progress of the emotional support that the device also provides.	Evaluate and focus on forms of user support, both physical and emotional. Evaluate the use of the device at an average time.
[78]	From the study by [79], the authors use a framework of processes and artifacts to identify the domains of augmented intelligence being these: memory, motivation, decision making and mood.	The authors propose a theoretical framework that provides a structural basis for exploring increased intelligence in the proposed domains.
[80]	The authors present a device to improve the posture of a human at a low cost, by using spring actuators of variable rigidity.	It is proposed to perform design optimization to allow compact and portable implementation of variable stiffness spring actuators.
[81]	The authors propose an augmented intelligence assistance system that captures the behavior of experienced personnel, with the objective of supporting the training of novice personnel in a company, avoiding that experienced employees use time in this training.	Carry out the evaluation of the training process using the Augmented Intelligence Assistant.
[82]	The authors propose a device capable of generating augmented reality cartography using Hololens glasses, this work can be collaborative because sub maps can be generated so that different users can build new maps. The created device is composed by the interaction of three agents: a robot, a human operator, and an augmented reality head mounted display.	The authors propose an online solution that allows merging of maps built by a robot, augmented reality head mounted display and a human operator, and the system could be integrated in any method solution that uses occupancy grids as cost maps for navigation.
[83]	The authors present an application to contact other people in case of an emergency, the increased humanity they applied is the power to amplify the view through cameras and increase the voice through speakers.	Extend the options of increased humanity that can be provided to users. Evaluate the usability and accessibility of the application by expert users.

Supplementary Material S1: Proposals for future work (cont.)

Article	About the paper	Future works
[84]	The authors perform the analysis of user behavior when searching through a tele-operation platform. To increase human capabilities, they created robots capable of helping to better visualize the user.	Make evaluations about the searches with more users.
[47]	The authors discuss the fields and terms related to AH, indicating that the fields are augmented senses, augmented action and augmented cognition.	The authors propose to work in the future on: Sensing technologies, Multisensory presentation technologies, Human activity measurement technologies, Actuation technologies and Ubiquitous information services and artificial intelligence technologies.
[85]	The authors present a neural network with a memory module to support humans in remembering concepts, this was done by training and learning to imitate the assimilation of simple concepts from humans using the deep reinforcement learning algorithm.	It is proposed to improve the tasks of formation of multimodal concepts, in the hope of contributing a step towards greater general intelligence.
[86]	The author developed a device to perform transcutaneous electrical stimulation of the distal-tibial nerve that can improve postural balance in the hands and feet with the aim of increasing tactile feedback from humans without the need for sight.	Add subjects to increase statistical power and compare the effect of transcutaneous electrical stimulation with existing training based on visual feedback.
[87]	The authors made a virtual representation of an arm equipped for the user to better perform his activities in such a way that a process of human-machine collaboration was generated.	From the evaluations obtained, the authors indicate that work should be done in the verbal context of communication with the proposed virtual arm.
[88]	A context-sensitive assistance application (visibility control, progress control, attention control, attention control and feedback) in augmented reality is proposed, this application was developed with Hololens.	It is proposed to improve the developed software architecture focusing on performance parameters. The authors also propose to integrate other types of assistance.
[89]	The authors propose possible ideas of developing different applications based on the terminology of transhumanization, for example: they include conflicts between physical and e-identities, introverting effects of in-body conversational agents, lack of harmony between AI and human minds, the challenge of producing inclusive games for distinctly-abled children and degradation of in-family social interactions due to the lack of need for information exchange.	This paper has the potential to encourage researchers and designers to critically reflect on issues that are usually overlooked when designing technologies, therefore, the authors propose to focus on the different areas of study and application development focused on the transhumanization theme.
[90]	The authors seek to raise awareness of the possibilities of augmented reality to introduce virtual animals for social, entertainment and educational purposes by augmenting physical environments, for example: one can use recordings of real animals, their behavior and movements, or digitally generated animated and naturally behaving virtual animals(AR) added to a physical environment.	Some proposals are: Optimize the visualization of augmented animals in an AR environment. Provide awareness of augmented animals, perception of urban furniture, behavior and interaction with passers-by.
[91]	The author presents related work based on Virtual/Mixed/Augmented Reality (VR/MR/AR) and reflects on how it can be used as a premise for Augmented Human research and the design of new human-machine systems.	The author proposes to prioritize key focus areas such as knowledge-intensive manual labor, e.g., knowledge-intensive maintenance and emerging augmented human technologies.
[92]	The authors developed a wearable proof-of-concept system capable of analyzing whether an argument or assertion is exposed with supporting evidence or not. Through the impact of argumentation mining and explainability of the AI feedback on the user through an experimental study of verbal statement evaluation tasks.	Consider developing a future system should also be capable of identifying the speech-act (argumentative, expressive act etc.) of an utterance to deliver relevant feedback. In future reasoning devices, a broader implementation of different types of logic (deductive, inductive, abductive) could be leveraged for greater real-life use.
